# Necrotizing Fasciitis and Streptococcal Toxic Shock Syndrome: A Case Report

**DOI:** 10.7759/cureus.73917

**Published:** 2024-11-18

**Authors:** Raffaele Merola, Clara De Negri, Angelina Merola, Alfonso Farina, Rosaria Antonietta Orlando, Alberto Pasqualucci, Alan D Kaye, Giustino Varrassi, Sahar Shekoohi, Pasquale De Negri

**Affiliations:** 1 Department of Neurosciences, Reproductive and Odontostomatological Sciences, University of Naples Federico II, Naples, ITA; 2 Department of Emergency, Università Statale degli Studi di Milano, Milan, ITA; 3 Department of Emergency, Sant’Anna and San Sebastiano Hospital, Caserta, ITA; 4 Department of Anesthesia and Critical Care, University of Perugia, Perugia, ITA; 5 Department of Anesthesiology, Louisiana State University Health Sciences Center, Shreveport, USA; 6 Department of Pain Medicine, Fondazione Paolo Procacci, Rome, ITA; 7 Department of Anesthesia, Intensive Care, and Pain Medicine, Sant’Anna and San Sebastiano Hospital, Caserta, ITA

**Keywords:** gas, group a streptococcus, necrotizing fascitis, streptococcical, toxic shock syndrome

## Abstract

Group A *Streptococcus *(GAS), particularly *Streptococcus pyogenes *(*S. pyogenes*), is a significant human pathogen responsible for infections often ranging from mild superficial conditions to severe, life-threatening diseases like necrotizing fasciitis (NF) and streptococcal toxic shock syndrome (STSS). This case report details the rapid deterioration of a previously healthy 49-year-old woman who presented with localized symptoms in her left thigh, later escalating to septic shock and multi-organ failure related to GAS infection. Initial evaluations indicated significant inflammation and acute kidney injury, prompting broad-spectrum antibiotic treatment. Despite aggressive management and transfer to the intensive care unit, the patient succumbed to complications of STSS and NF. An autopsy confirmed systemic dissemination of *S. pyogenes*. This case underscores the urgent need for heightened clinical awareness and timely intervention in patients with rapid onset of severe infections, emphasizing the critical importance of public health initiatives to mitigate GAS-related morbidity and mortality. Future research should focus on understanding the pathophysiology and risk factors associated with severe GAS infections to develop targeted therapies.

## Introduction

The Group A* Streptococcus* (GAS), or *Streptococcus pyogenes* (*S. pyogenes*), an obligate human pathogen, is one of the top infectious causes of human mortality and is responsible for more than 500,000 annual deaths worldwide [1]. Most infections, more than one billion annually, are superficial and relatively mild, such as impetigo and pharyngitis. However, these infections trigger immune sequelae such as rheumatic heart disease [2]. Annually, millions of these mild infections develop into severe diseases, including bacteremia, cellulitis, puerperal sepsis, necrotizing fasciitis (NF), and streptococcal toxic shock syndrome (STSS) [3].

NF is a rapidly progressive, life-threatening soft tissue infection characterized by necrosis of fascial planes and surrounding structures [4]. Typically, the infection originates from a leak in the skin, such as wounds, surgical incisions, or even small abrasions, allowing opportunistic pathogens to invade the deep fascial layers [5]. This clinical condition represents a difficult clinical challenge due to its aggressive nature, which leads to high morbidity and mortality rates despite advances in clinical care [4]. The disease progresses rapidly, sometimes suddenly, and delayed treatment can cause widespread tissue destruction and systemic complications [6].STSS is characterized by hypotension and multiple organ failure (MOF), often with immunological manifestations such as rash [7]. STSS is thought to be triggered in part by superantigens and other bacterial virulence factors. Furthermore, STSS is associated with substantial morbidity, with most cases requiring intensive care admission, with a mortality rate of more than 25% in the first 24 hours [8,9]. We, therefore, present a case of STSS in a 49-year-old female patient admitted to the emergency department of Sant'Anna and San Sebastiano Hospital in Caserta, Italy.

## Case presentation

This present case involves a 49-year-old female patient admitted to the emergency department for a rash on the medial aspect of her left thigh in the context of worsening pain symptoms in the same area and in the ipsilateral inguinal region that had started three days before the onset of the rash and admission to the emergency department. On remote pathological history, the patient reported only arterial hypertension and psoriasis. On physical examination, the patient presented with swelling and reddening of the medial aspect of the left thigh and pain on palpation both contextually and in the ipsilateral inguinal region. Blood samples were taken for testing, which showed increased C-reactive protein, myoglobin, creatinine, and d-dimer (Table 1).

**Table 1:** Blood test results

laboratory parameters	Patient values	Reference range
C-reactive protein	Increased (35.78 mg/dl)	<1.0 mg/dL
Myoglobin	Increased (133.60 ng/ml)	25 to 72 ng/mL
Creatinine	Increased (1.3 mg/dl)	0.6-1.1 mg/dL for females, 7-1.3 mg/dL for males
D-dimer	Increased (803 ng/ml)	Undetectable or only detectable at a very low level

An ultrasound examination of the area of clinical interest was performed, which showed extensive and marked thickening of the subcutis, with contextual, fluid stratum extending to the superficial fascial planes unharmed; the underlying muscular compartment was also normal. Multiple reactive lymph nodes were evident in the inguinal area. The findings, therefore, depicted an infectious process. The patient was given ketorolac 30 mg to relieve pain symptoms with modest benefit. Given the clinical picture and laboratory-instrumental findings, the patient refused admission, antibiotic therapy, and infectious, dermatological, and vascular surgery examinations were recommended, and she was referred to her general practitioner.

**Figure 1:**
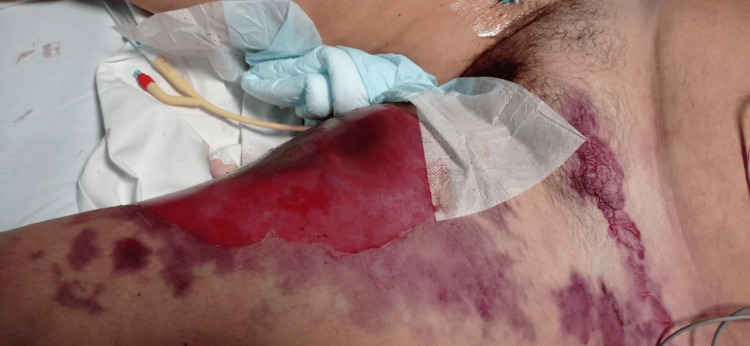
The appearance of hemorrhagic bullous lesions on the medial side and in the inguinal area of the left thigh

**Figure 2:**
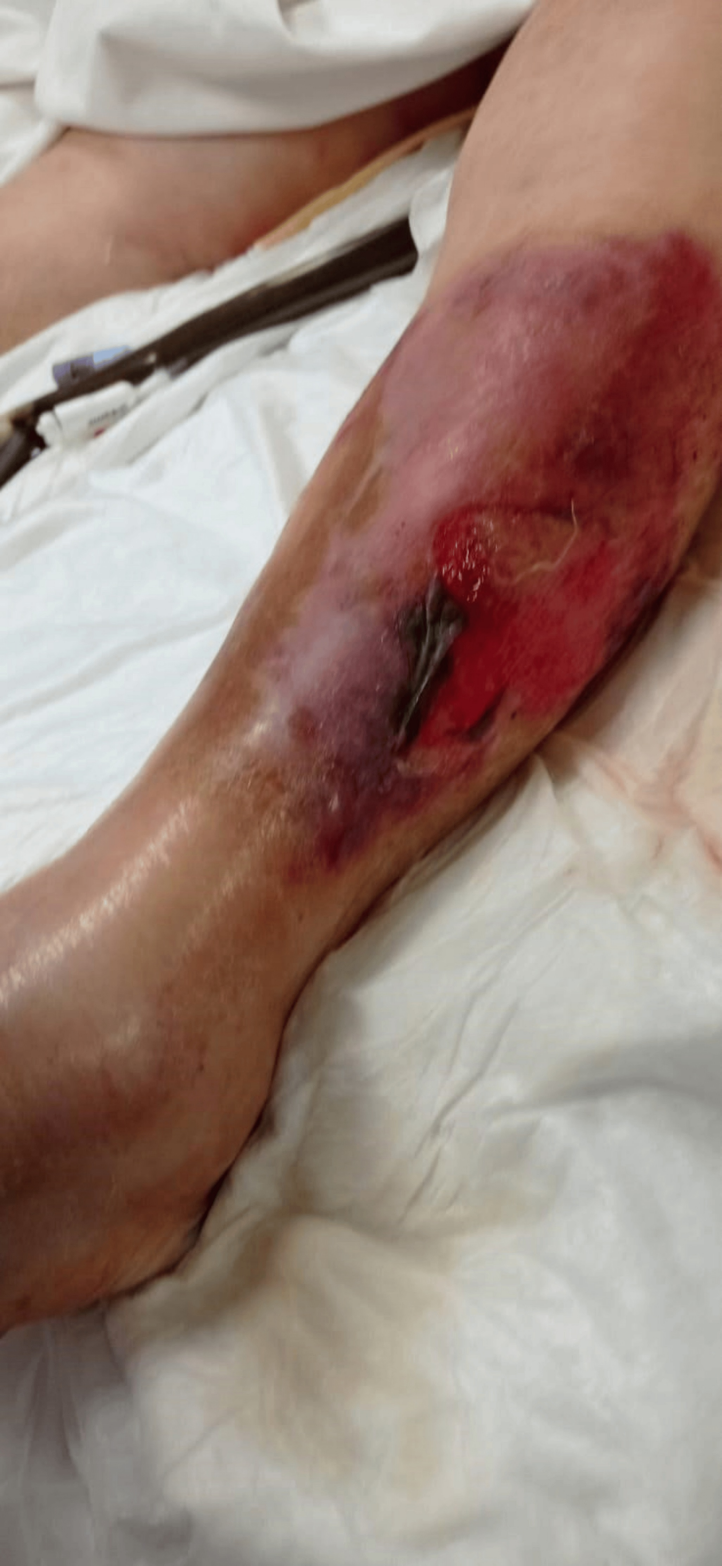
The appearance of a necrotic hemorrhagic lesion on the lateral side of the ipsilateral leg

Approximately 12 hours after discharge, the case was readmitted to the emergency department due to worsening of the clinical picture, with increasingly intense pain, spread of the rash with the appearance of hemorrhagic bullous lesions on the medial side and in the inguinal area of the left thigh and the appearance of a necrotic hemorrhagic lesion on the lateral side of the ipsilateral leg (Figures 1-2) and dyspnea. On admission to the emergency department, the patient was neurological, hemodynamic, and respiratory stable, with a Glasgow Coma Scale (GCS) of 15, blood pressure (BP) of 110/60 mmHg, heart rate of 108 beats per minute, respiratory rate of 24 beats per minute, and arterial oxygen saturation (SaO2) of 98%.

She underwent new blood examinations that showed further increases in C-reactive protein, creatinine, and d-dimer, hemoglobin of 10.9 g/dl, neutrophilia with normal white blood cells, glycemia, azotemia, calcium of 6. 8 mg/dl, hypoproteinemia and hypoalbuminemia, increased transaminases (aspartate transaminase (AST) and alanine transaminase (ALT)), procalcitonin (PCT), decreased prothrombin time (PT), international normalised ratio (INR), activated partial thromboplastin time (aPTT), fibrinogen of 633 mg/dl, and markedly decreased antithrombin III (Table 2).

**Table 2:** Blood test results approximately 12 hours after discharge INR: International normalized ratio; AST: aspartate transaminase; ALT: alanine transaminase

laboratory parameters	Patient values	Reference range
C-reactive protein	36.51 mg/dl	<1.0 mg/dL
Creatinine	2.6 mg/dl	0.6-1.1 mg/dL for females, 7-1.3 mg/dL for males
D-dimer	928 ng/ml	Undetectable or only detectable at a very low level
Hemoglobin	10.9 g/dl	12.0 to 15.5 g/dL for females, 13.5 to 17.5 g/dL for males
Neutrophilia with normal white blood cells	78.7% of the leucocyte formula	A normal neutrophil level is 1,450 to 7,500 neutrophils per microliter
Glycemia	68 mg/dl	70 to 99 mg/dL
Azotemia	36 mg/dl	8-20 mg/dL
Calcium	6. 8 mg/dl	8.5 to 10.5 mg/dl
Hypoproteinemia	5.0 g/dl	6.0 and 8.3 (g/dL)
Hypoalbuminemia	2.2 g/dl	3.5 to 5.5 (g/dL)
AST	355 IU/L	0 to 35 IU/L
ALT	140 IU/L	0 to 45 IU/L
Procalcitonin (PCT)	31.50 ng/ml	<0.1 μg/L
Prothrombin time (PT), INR	1.74	11 to 13.5 seconds
Activated partial thromboplastin time (aPTT)	45 seconds	Between 25 and 35 seconds
Fibrinogen	633 mg/dl	200-400 mg/dl

**Figure 3:**
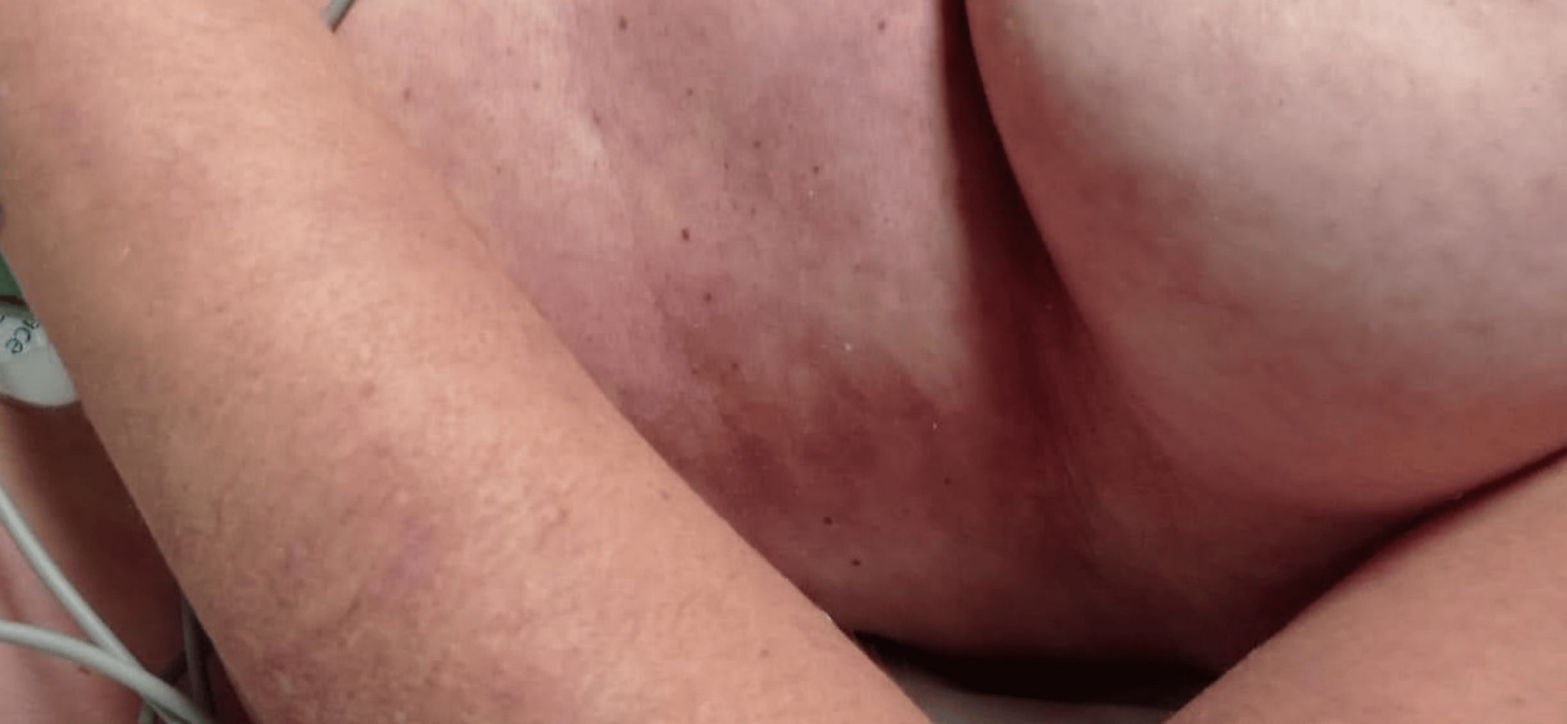
Skin marbling in the thoracic region

Samples were also taken for culture tests, which turned out to be negative. On blood analytical examination, metabolic acidosis was found (pH, 7.23; PaCO_2_, 19 mmHg; HCO_3_, 8.0 mmol/L; Lac, 9.8 mmol/L); there was no impairment of pulmonary exchange function (PaO_2_ > 300 mmHg during oxygen therapy with 50% Venturi mask). The case was admitted to the emergency medicine ward with a diagnosis of fasciitis of the left lower limb and was started on antibiotic therapy with meropenem (Merrem), vancomycin and anidulafungin, volume filling, and correction of acidosis with isotonic saline and sodium bicarbonate (100 mEq/L). Despite the therapeutic measures taken, there was a progressive and rapid deterioration in the patient's clinical condition. About an hour after being admitted to the emergency medicine ward, the patient presented a clinical picture of septic shock, with a confusional state, marked hypotension (65/40 mmHg), tachycardia (heart rate 120 beats per minute), tachypnea (respiratory frequency > 35 beats per minute), and anuria. The resuscitating doctor was then alerted, who, in view of the clinical picture, advocated immediate transfer to the intensive care unit (ICU). The blood gas analysis on admission to the ICU revealed very severe metabolic acidosis and marked hypoxemia despite manual ventilation with 100% FiO_2_ (pH < 7.00, HCO_3_^-^ incalculable, Lac of 14.9 mmol/L, PaO_2_ of 41 mmHg). Clinical examination also revealed widespread skin marbling on all four limbs and in the thoracic region (Figure 3) and hypothermia (core temperature <35°C).

Therefore, after sedation and neuromuscular blocker paralysis, the patient was intubated and subjected to invasive mechanical ventilation. After cannulation of a central venous vessel, volume filling was carried out with crystalloid solutions (isotonic physiological saline and ringer lactate) at a rate of 30 ml/Kg in the first hour, sodium bicarbonate 200 mEq/L was administered to try to correct the acidosis, corticosteroid therapy was started, and the patient was warmed up using a thermal blanket. The circulation was supported by noradrenaline in continuous infusion (0.8 micrograms/kg/min). Approximately three hours after the patient's admission to the ICU, the hemodynamic picture improved with a heart rate of 110 beats per minute, BP of 118/50 mmHg, and SpO_2_ of 100%; despite this and the improvement in pulmonary exchange function (PaO_2_ of 347 mmHg in mechanical ventilation with FiO_2_ at 70%, PaCO_2_ of 41 mmHg), the severe metabolic acidosis persisted (pH 7.02, Lac > 17 mmol/L, HCO_3_^-^ of 10.6) and anuria despite stimulation with furosemide. After two hours of apparent stability, there was an episode of severe bradycardia (HR: 30 bpm), promptly treated with 1 mg of atropine bolus; blood gas analysis showed a metabolic picture substantially unchanged from the previous one and severe hyperpotassemia (pH < 7.00, HCO_3_^-^ incalculable, Lac > 17 mmol/L, K+ 9.5 mmol/L), which was treated by administering 1 g of calcium chloride. Diffusion of the marbling to the four limbs was also observed. Ten minutes after the bradycardia episode, asystole ensued, which persisted despite the advanced cardiopulmonary resuscitation maneuvers carried out, and therefore, death was established.

In view of the fulminating development of the clinical picture, an autopsy was requested. The autopsy revealed the presence of *S. pyogenes* in the pleural and pericardial fluid. This finding, combined with the morphological features found, led to the conclusion that the patient's death was related to MOF secondary to NF, STSS, and disseminated intravascular coagulation (DIC).

## Discussion

The present case report highlights the rapid and devastating course of STSS secondary to NF caused by GAS in a previously healthy 49-year-old female. The case exemplifies the aggressive nature of GAS infections, which, although commonly associated with mild superficial conditions, can escalate quickly into life-threatening illnesses like NF and STSS [2]. The patient's initial presentation was characterized by localized symptoms in the left thigh, which included pain, swelling, and the development of a rash. Laboratory findings indicated significant inflammation, evidenced by elevated CRP and d-dimer levels, alongside signs of acute kidney injury and metabolic disturbance. These findings are consistent with the inflammatory response triggered by invasive GAS infections, which often elicit a robust immune reaction that can lead to systemic complications [10]. Despite initial outpatient management, the patient's clinical condition deteriorated rapidly, illustrating a critical challenge in the timely identification and treatment of STSS. The progression from localized infection to septic shock within a short timeframe underscores the importance of vigilant monitoring and the need for early intervention [11].

The initial negative culture results do not negate the diagnosis; the rapid progression and clinical picture are often sufficient for initiating broad-spectrum antibiotics, as was appropriately done upon readmission [12]. The patient's eventual transfer to the ICU was a necessary step given the signs of septic shock, including hypotension, altered mental status, and persistent metabolic acidosis. The management strategies, such as aggressive fluid resuscitation, mechanical ventilation, and inotropic support, reflect current best practices for managing severe STSS [13]. However, despite these interventions, the patient succumbed to MOF, which is a stark reminder of the high mortality rates associated with this condition, particularly when not addressed swiftly [8,9].

The autopsy findings confirmed the presence of *S. pyogenes* in the pleural and pericardial fluid, indicating systemic dissemination of the bacteria and supporting the diagnosis of STSS and NF. This outcome illustrates the critical need for healthcare providers to be aware of the potential for rapid progression of GAS infections, especially in patients presenting with unexplained skin changes and systemic signs of disease. This case also emphasizes the importance of public health initiatives to reduce the incidence of GAS infections and the subsequent complications that can arise from them.

## Conclusions

Educational efforts to raise awareness about the signs and symptoms of severe infections, along with strategies for early intervention, could play a crucial role in improving outcomes for affected patients. This case report highlights the need for a strong clinical suspicion of STSS in patients with acute localized disease who develop systemic symptoms so rapidly. It also highlights the necessity for rapid diagnostic and therapeutic measures to improve survival rates in patients with NF and STSS. Future research should focus on understanding the pathophysiology of GAS infections, identifying risk factors for severe disease, and developing targeted therapies to mitigate the associated morbidity and mortality.
